# Parent and carer experiences of health care professionals’ communication about childhood obesity: a qualitative systematic review protocol

**DOI:** 10.11124/JBIES-22-00017

**Published:** 2022-09-05

**Authors:** Terhi Koivumäki, Maria Kääriäinen, Anna-Maria Tuomikoski, Marja Kaunonen

**Affiliations:** 1 Health Sciences, Faculty of Social Sciences, Tampere University, Tampere, Finland; 2 Finnish Centre for Evidence-Based Health Care: A JBI Centre of Excellence, Helsinki, Finland; 3 Research Unit of Nursing Science and Health Management, University of Oulu, Oulu, Finland; 4 Oulu University Hospital, Finland; 5 General Administration, Pirkanmaa Hospital District, Tampere, Finland

**Keywords:** childhood obesity, communication, experience, health care professionals, parents/carers

## Abstract

The objective of the systematic review is to identify, critically appraise, and synthesize the best available qualitative evidence on parent and carer experiences of health care professionals’ communication about childhood obesity.

**Introduction:**

Parents and carers play a key role in a child’s environment and healthy development, which is why they can find it confronting to discuss their child’s weight. This review will provide an insight into the experiences of parents and carers with health care professionals’ communication about their child's overweight or obesity.

**Inclusion criteria:**

This qualitative review will consider participants who are parents and carers with a child with overweight or obesity (birth to 12 years). The phenomenon of interest is parents’ and carers’ lived experiences of childhood obesity communication from a health care professional, and the context is health care settings. Communication includes verbal or written communication about a child's obesity from health care professionals received by a parent or carer.

**Methods:**

The proposed review will systematically search the following databases: MEDLINE (EBSCO), CINAHL (EBSCO), PsycINFO (Ovid), Scopus, LILACS, and the Finnish health sciences database MEDIC. ProQuest Dissertations and Theses (ProQuest) will be searched for unpublished articles. A manual search will supplement the database searches. The quality of included studies will be assessed independently by 2 reviewers, and the qualitative data will be extracted from papers by 2 independent reviewers using the standardized JBI data extraction tool. The recommended JBI approach to critical appraisal, study selection, data extraction, and data synthesis meta-aggregation will be used.

**Systematic review registration number:**

PROSPERO CRD42022297709

## Introduction

Childhood obesity is a major concern worldwide. In 2019, globally, 38 million children under the age of 5 were overweight or obese, and the prevalence among children and adolescents aged 5 to 19 with overweight has increased from 4% (1975) to 18% (2016). Children under 5 years of age are defined as overweight if their weight-for-height is greater than 2 standard deviations above the WHO Child Growth Standards median, and are defined as obese when their weight-for-height is greater than 3 standard deviations. Children aged between 5 and 19 years are defined as overweight when their BMI-for-age is greater than 1 standard deviation above the WHO Growth Reference median and defined as obese when their BMI-for-age is greater than 2 standard deviations.^1^ There have been many changes to children’s environments over the past decades (such as changes in the food environment, time use and built environment, increased food supply, increased screen time, and decreased physical activity) that have increased energy intake and decreased the expenditure of energy.^2^ The increase in childhood obesity cannot be explained by a change in genetics; rather, it is directly influenced by changes in children’s eating and physical activity behaviors that change the energy balance.^3^

When treating childhood obesity, targeting parents is an effective way to effect change.^4^ Health care professionals (HCPs) describe childhood obesity as a difficult issue to communicate with parents about and feel that they do not always have enough knowledge and experience to do so.^5^-^7^ In addition, parents do not always recognize that their child is overweight.^8^-^12^ Discussing weight and obesity issues with parents is a health communication dilemma^13^ and more information is needed about the most effective communication strategies. Parents experience an emotional response when hearing about their child’s weight,^14^,^15^ which can affect the way they receive the communication. Feedback on a child’s weight increases parents’ ability to recognize that their child is overweight; however, the impact of weight feedback on behavior change is limited and more knowledge is needed to identify ways to communicate effectively with parents.^10^

There are multiple reasons why parents may hesitate or avoid talking about their child’s weight. Parents of children with obesity may fear that their child could experience harm during treatment for the obesity, and parents may also worry that they will be accused of causing the condition.^16^ Parents’ negative experiences with their own body weight can lead to a decision not to discuss body weight issues with the child.^17^ The way HCPs communicate with parents has a major role in motivating them. Parents are better motivated when their focus is on their child’s well-being and happiness than on risks attached to obesity.^8^ Parents want to be confident that communication about childhood obesity is non-judgmental^16^ and that it will be addressed in a sensitive and respectful manner.^9^ Parents may reject information about their child being overweight, feel the information lacks credibility, or that it should be targeted to other parents rather than themselves.^18^

When identifying children’s obesity, the HCP is advised to communicate with the family sensitively, including being careful with their choice of words, to minimize embarrassment or harm to the child’s self-esteem. The HCP should be supportive, empathetic, and non-judgmental.^3^ Parents want concrete, regular, timely, and individualized recommendations in a confidential and non-judgmental atmosphere. The communication should also be directed to the child on a level they can understand, so that the child is not frightened of the health consequences attached to obesity. The guidance should be done in a polite way and the words used should be easily understandable.^19^-^21^

A scoping review by McPherson *et al.* focused on the best ways for HCPs to communicate with children and their families about obesity and weight-related topics, and encompassed research conducted with HCPs, parents, and children. The results emphasize including all stakeholders in discussions, early and regular communication, strength-based language that emphasizes health over weight, collaborative goal-setting, and using appropriate tools and resources. The review concluded that best practices related to evidence-based, weight-related communication are lacking.^22^

Most often, communication about a child’s weight takes place during a meeting with HCPs in primary care or pediatric weight management care. Communication can be conducted through interactive visual presentations,^23^ telephone counseling,^24^ with a letter,^25^ or other written feedback.^26^ The format of the feedback does not play a major role in parents attending further treatment, but parents do have clear preferences for the format, timing, content, and amount of information they want to receive, and also clear preferences on how HCPs should communicate with them or with the child.^14^ It should also be considered whether the child should be present during discussions about their weight.^9^ While there are several studies on communication about childhood obesity, research concentrating on parents’ and carers’ experiences is scarce. The objectives of this review are to explore parents’ and carers’ experiences of communication with HCPs in primary and secondary care when discussing a child with overweight or obesity.

A preliminary search of PROSPERO and *JBI Evidence Synthesis* was conducted and no current or in-progress systematic reviews on the topic were identified. There were 25 results in PROSPERO with search phrases containing parents AND communicating, but none of them focused on counseling for childhood obesity. From *JBI Evidence Synthesis*, there were 17 results for the search words obesity AND communicat^∗^ AND parent^∗^, but none of those were in the childhood obesity communication context. There is a need to synthesize separate studies on parents’ and carers’ perceptions on communicating about childhood obesity.

## Review question

What are parents’ and carers’ experiences with HCPs’ communication when their child is overweight or obese?

## Inclusion criteria

### Participants

This qualitative review will consider studies that include mothers, fathers, or other carers of a child aged from birth to 12 years who is overweight or obese.

### Phenomena of interest

This review will consider studies that describe parents’ or carers’ experiences of communication with HCPs about childhood obesity. Communication includes verbal or written communication about a child’s obesity from the HCP, received by a parent or carer of a child with overweight or obesity.

### Context

The review will consider studies that have been conducted in a health care setting (primary or secondary care) where the main reason for the consultation is prevention or care for childhood overweight or obesity.

### Types of studies

This review will consider studies that focus on qualitative data, including, but not limited to, designs such as phenomenology, grounded theory, ethnography, action research, and mixed methods. Descriptive qualitative studies that describe the experiences of parents and carers will also be considered. Quantitative studies (eg, meta-analyses), editorials, commentaries, letters, and conference abstracts will be excluded. Studies published from 2010 will be included, as McPherson *et al.* discovered that the majority of articles about obesity communication were published in or after 2010.^22^

## Methods

The proposed systematic review will be conducted in accordance with the JBI methodology for systematic reviews of qualitative evidence using the JBI critical appraisal checklist for qualitative research from the JBI System for the Unified Management, Assessment and Review of Information (JBI SUMARI; JBI, Adelaide, Australia).^27^

### Search strategy

An initial limited search of the CINAHL, PROSPERO and *JBI Evidence Synthesis* databases was undertaken to identify articles on the topic, and a full search strategy was developed for CINAHL (EBSCO; see Appendix I). The search strategy will aim to locate both published and unpublished studies. The databases to be searched include MEDLINE (EBSCO), CINAHL (EBSCO), PsycINFO (Ovid), Scopus, LILACS, and the Finnish health sciences database MEDIC. Sources of unpublished studies and gray literature to be searched include ProQuest Dissertations and Theses. When conducting the search, assistance from an information specialist will be utilized, and this help was also used when conducting the initial search for this protocol. The search strategy, including all identified keywords and index terms, will be adapted for each database and/or information source. The reference list of all included sources of evidence will be screened for additional studies.

This review will consider studies from all geographic settings and there will be no language limits. To manage studies beyond the authors’ competence (languages other than English, Swedish, Finnish, German, French, and Spanish), the English abstract will be carefully examined, and all studies that include the inclusion criteria will be translated into English with the help of translation programs, by contacting the authors to gain the information needed, or by asking for assistance from other JBI Centers of Excellence (https://jbi.global/global-networks/collaboration).

### Study selection

Following the search, all identified citations will be collated and uploaded into Covidence (Veritas Health Innovation, Melbourne, Australia) and duplicates removed. Following a pilot test, titles and abstracts will be screened by 2 or more independent reviewers for assessment against the inclusion criteria for the review. Potentially relevant studies will be retrieved in full and their citation details imported into JBI SUMARI.^27^ The full text of selected citations will be assessed in detail against the inclusion criteria by 2 or more independent reviewers. Reasons for the exclusion of papers at full text that do not meet the inclusion criteria will be recorded and reported in the systematic review. Any disagreements that arise between the reviewers at each stage of the selection process will be resolved through discussion or with additional reviewers. The results of the search and the study inclusion process will be reported in full in the final systematic review and presented in a Preferred Reporting Items for Systematic Reviews and Meta-Analyses (PRISMA) flow diagram.^28^

### Assessment of methodological quality

Eligible studies will be critically appraised by at least 2 independent reviewers for methodological quality using the standard JBI critical appraisal checklist for qualitative research.^27^ Authors of papers will be contacted to request missing or additional data for clarification, where required. Any disagreements that arise between the reviewers will be resolved through discussion or with a third reviewer. The results of the critical appraisal will be reported in narrative format and in a table. All studies, regardless of their methodological quality, will undergo data extraction and synthesis (where possible).

### Data extraction

Data will be extracted from studies included in the review by 2 independent reviewers using the standardized JBI data extraction tool and the JBI critical appraisal checklist for qualitative research from JBI SUMARI.^27^ The data extracted will include specific details about the populations, context, culture, geographical location, study methods, and the phenomena of interest relevant to the review objective. Findings and their illustrations will be extracted and assigned a level of credibility.

### Data synthesis

Qualitative research findings will, where possible, be pooled using JBI SUMARI with the meta-aggregation approach.^27^ This will involve the aggregation or synthesis of findings to generate a set of statements that represent that aggregation, through assembling the findings and categorizing the findings on the basis of similarity in meaning. These categories will then be synthesized in order to produce a single comprehensive set of synthesized findings that can be used as a basis for evidence-based practice. Where textual pooling is not possible, the findings will be presented in narrative format. Only unequivocal and credible findings will be included in the synthesis.

### Assessing confidence in the findings

The final synthesized findings will be graded according to the ConQual approach for establishing confidence in the output of qualitative research synthesis and presented in a Summary of Findings.^29^ The Summary of Findings will include the major elements of the review and detail how the ConQual score was developed. Included in the Summary of Findings will be the title, population, phenomena of interest, and context for the specific review. Each synthesized finding from the review will then be presented, along with the type of research informing it, a score for dependability and credibility, and the overall ConQual score.

## Acknowledgments

Information Specialist Jaana Isojärvi for assistance with the search strategy for this protocol.

## Author contributions

TK, MKä, A-MT, MKa designed the analysis and search strategy, collected data, performed the analysis, and wrote the manuscript. TK performed the search strategy.

## Appendix I: Search strategy

### CINAHL (EBSCO)

Date searched: December 17, 2021

**Table:**

Search #	Terms	Results retrieved
1.	(MH “Pediatric Obesity”)	16,011
2.	(MH “Obesity”) AND ((MH “Child”) OR (MH “Infant”) OR (MH “Child, Preschool”))	12,876
3.	TI ((obesity OR obese OR overweight) AND (child or children or childhood or pediatric or paediatric)) OR AB ((obesity OR obese OR overweight) AND (child or children or childhood or pediatric or paediatric))	25,155
4.	S1 OR S2 OR S3	36,049
5.	MH (communication)	87,003
6.	TI (communicat^∗^ OR conversation^∗^ OR discuss^∗^ OR letter^∗^ OR feedback OR telephone counsel^∗^ OR dialog^∗^) OR AB communicat^∗^ OR conversation^∗^ OR discuss ^∗^OR dialog^∗^ OR letter^∗^ OR feedback OR telephone counsel^∗^ OR dialog^∗^)	282,682
7.	S5 OR S6	326,728
8.	MH parents OR MH caregivers OR MH mothers OR MH fathers	120,014
9.	TI (parent^∗^ OR caregiver^∗^ OR mother^∗^ OR father^∗^) OR AB (parent^∗^ OR caregiver^∗^ OR mother^∗^ OR father^∗^)	280,714
10.	S8 OR S9	316,961
11.	S7 AND S10	28,645
12.	MH qualitative studies OR MH phenomenological research OR MH Ethnographic research OR Grounded theory or MH interviews OR MH thematic analysis OR MH focus groups OR MH narratives OR MH metasynthesis OR MH action research	306,996
13.	TI (focus group^∗^ OR qualitative OR descriptive OR ethnograph^∗^ OR fieldwork OR field work OR key informant OR grounded theory OR phenomenolo^∗^ OR metasynthesis OR meta synthesis OR meta-synthesis OR action research OR mixed methods) OR AB (focus group^∗^ OR qualitative OR descriptive OR ethnograph^∗^ OR fieldwork OR field work OR key informant OR grounded theory OR phenomenolo^∗^ OR metasynthesis OR meta synthesis OR meta-synthesis OR action research OR mixed method^∗^)	298,413
14.	TI (semi structured OR semistructured OR unstructured OR informal OR indepth OR in-depth OR “face to face”) OR AB (semi structured OR semistructured OR unstructured OR informal OR indepth OR in-depth OR “face to face”)	135,441
15.	S12 OR S13 OR S14	514,216
16.	S4 AND S11 AND S15	199
